# Supplementary material for: Naturally Occurring Fusarium Species and Mycotoxins in Oat Grains from Manitoba, Canada
Source: Toxins (Basel). 2021 Sep 18;13(9):670. doi: 10.3390/toxins13090670 (PMC8473195; doi:10.3390/toxins13090670)
Supplement: Supplementary file 1 [file toxins-13-00670-s001.zip › toxins-1381308-SI.pdf]

Supplementary Materials: Naturally Occurring *Fusarium* Species and Mycotoxins in Oat Grains from Manitoba, Canada

M. Nazrul Islam, Mourita Tabassum, Mitali Banik, Fouad Daayf, W. G. Dilantha Fernando, Linda J. Harris, Srinivas Sure and Xiben Wang

Table S1. Fusarium species/chemotype-specific primers sequences.

Target *Fusarium* spp.	Name of primer	Sequences (5'-3')	References
**A.** Conventional PCR (identification)			
F. poae	Fp82F	CAAGCAAACAGGCTCTTCACC	
	Fp82R	TGTTCCACCTCAGTGACAGGTT	
F. graminearum	Fg16F	CTCCGGATATGTTGCGTCAA	
	Fg16R	GGTAGGTATCCGACATGGCAA	
F. sporotrichioides	FspF	CGCACGTATAGATGGACAAG	Demeke et al. (2005)
	FspR	GTCAGAAGAGACGCATCCGCC	
F. avenaceum	FaF	CAAGCATTGTCGCCACTCTC	
	FaR	GTTTGGCTCTACCGGGACTG	
F. culmorum	FcoF1	ATGGTGAACTCGTCGTGGC	
	FcoR1	CCCTTCTTACGCCAATCTCG	
**B.** RT-qPCR			
F. poae	FpA51F	ACCGAATCTCAACTCCGCTTT	
	FpA98R	GTCTGTCAAGCATGTTAGCACAAGT	
F. graminearum	FgB397F	CCATTCCCTGGGCGCT	Nicolaisen et al. (2009)
	FgB411R	CCTATTGACAGGTGGTTAGTGACTGG	
F. sporotrichioides	FspA18F	GCAAGTCGACCACTGTGAGTACA	
	FspA85R	CTGTCAAAGCATGTCAGTAAAAATGAT	
**C.** Chemotype detection			
Multiplex PCR			
F. graminearum	3CON	TGGCAAAGACTGGTTCAC	
	3NA	GTGCACAGAATATACGAGC	
	3D15A	ACTGACCCAAGCTGCCATC	Ward et al. (2008)
	3D3A	CGCATTGGCTAACACATG	
Singleplex PCR			
F. poae	nivPF	TATCCTTGCATGGCAATGCC	
	nivPR	AAATGGCGATACGAGTATTGA	Dinolfo et al. (2012)
**D.** Phylogenetic analysis			

F. poae	TEF1αF	ATGGGTAAGGAGGAGAAGACT	
	TEF1αR	GGAAGTACCAGTGATCATGTT	
	Tri1F	CGGGCCTGTGGACATCT	Witte et al. (2021)
	Tri1R	GGGTTTCTTGAGCCAATGGAAT	
	Tri8F	ACAACATCCTCTATCGCACAAC	
	Tri8R	GTGATATCCCATGATGCCTTCC	

Table S2 Precursor and product ions (m/z), retention times, and recoveries of various mycotoxins.

Mycotoxin (Abbreviation)	Precursor ion (m/z)	Retention Time (min)	Recoveries Mean ± SD[a]
Deoxynivalenol (DON)	297.1333	4.39	91 ± 7
Diacetoxyscirpenol (DAS)	384.2017	8.67	95 ± 6
Nivalenol (NIV)	313.1282	3.36	81 ± 6
Beauvericin (BEA)			47 ± 9
HT-2 Toxin (HT-2)	442.2435	9.76	94 ± 5
T-2 Toxin (T-2)	484.2541	10.54	119 ± 6
Moniliformin (MON)			
Enniatin A (ENN A)	699.4903	14.28	61 ± 6
Enniatin A1 (ENN A1)	685.4746	14.11	63 ± 7
Enniatin B (ENN B)	657.4433	13.68	55 ± 4
Enniatin B1 (ENN B1)	671.4903	13.91	57 ± 3

[a] Number of spiked samples, *n* = 9

Table S3 List of *F. poae* strains and corresponding crop districts/location of isolates and rotational crop varieties in Manitoba (2016-2018). Group designation was based on *Tef-1α-Tri1 - Tri8* phylogenetic analysis.

Isolate code	Group	Crop district	Previous crop
FP2016-27	I	CMB	Canola
FP2017-28		CMB	Canola
FP2016-24		CMB	Canola
FP2016-07		CMB	Canola
FP2016-28		CMB	Flax
FP2016-06		CMB	Canola
FP2016-05		CMB	Canola
FP2016-03		CMB	Canola
FP2016-02		CMB	Canola
FP2016-01		CMB	Canola
FP2016-69		EMB	Flax
FP2016-29		CMB	Flax
FP2018-15		SWMB	Canola
FP2017-12		SWMB	Canola
FP2018-07		CMB	Cereal
FP2016-70		CMB	Cereal
FP2017-32		CMB	Canola
FP2017-89		CMB	Canola
FP2018-89		CMB	Canola
FP2018-32		CMB	Canola
FP2017-70		CMB	Flax
FP2018-31		SWMB	Cereal
FP2018-39		SWMB	Canola
FP2018-74		SWMB	Canola
FP2018-108	II	INMB	Canola
FP2018-106		INMB	Canola
FP2018-105		INMB	Canola
FP2017-30		INMB	Canola
FP2018-101		INMB	Cereal

FP2016-30		INMB	Flax
FP2018-95		INMB	Cereal
FP2017-97		INMB	Cereal
FP2017-96		INMB	Canola
FP2018-40		NWMB	Canola
FP2018-88		NWMB	Canola
FP2018-84		NWMB	Canola
FP2016-61		NWMB	Cereal
FP2016-43		NWMB	Canola
FP2016-42		NWMB	Canola
FP2016-40		NWMB	Canola
FP2016-59		NWMB	Canola
FP2016-62		NWMB	Cereal
FP2016-71		EMB	Cereal
FP2018-104		INMB	Canola
FP2018-25		NWMB	Canola
FP2018-02		NWMB	Flax
FP2018-82		NWMB	Canola
FP2018-83		NWMB	Canola
FP2018-81		NWMB	Canola
FP2018-44		NWMB	Canola
FP2018-38	III	NWMB	Canola
FP2017-28		NWMB	Canola
FP2018-85		NWMB	Canola
FP2018-34		NWMB	Canola
FP2016-79		EMB	Canola
FP2017-77		EMB	Cereal
FP2017-74		CMB	Cereal
FP2017-51		SWMB	Cereal
FP2016-13		SWMB	Flax
FP2017-68		SWMB	Canola
FP2018-13		CMB	Canola
FP2017-88	IV	CMB	Flax
FP2018-17		CMB	Canola
FP2018-16		CMB	Canola
FP2018-08		CMB	Flax

FP2016-13	CMB	Flax
FP2016-08	CMB	Canola
FP2018-12	CMB	Canola
FP2017-46	SWMB	Canola
FP2017-50	SWMB	Canola
FP2017-54	SWMB	Canola
FP2017-56	SWMB	Canola
FP2017-63	SWMB	Canola
FP2017-66	SWMB	Canola
FP2017-71	SWMB	Flax
FP2017-18	SWMB	Canola
FP2017-17	SWMB	Canola
FP2018-94	INMB	Canola
FP2018-107	INMB	Canola
FP2016-88	CMB	Canola
FP2016-90	CMB	Canola
FP2018-112	INMB	Canola
FP2016-92	CMB	Canola

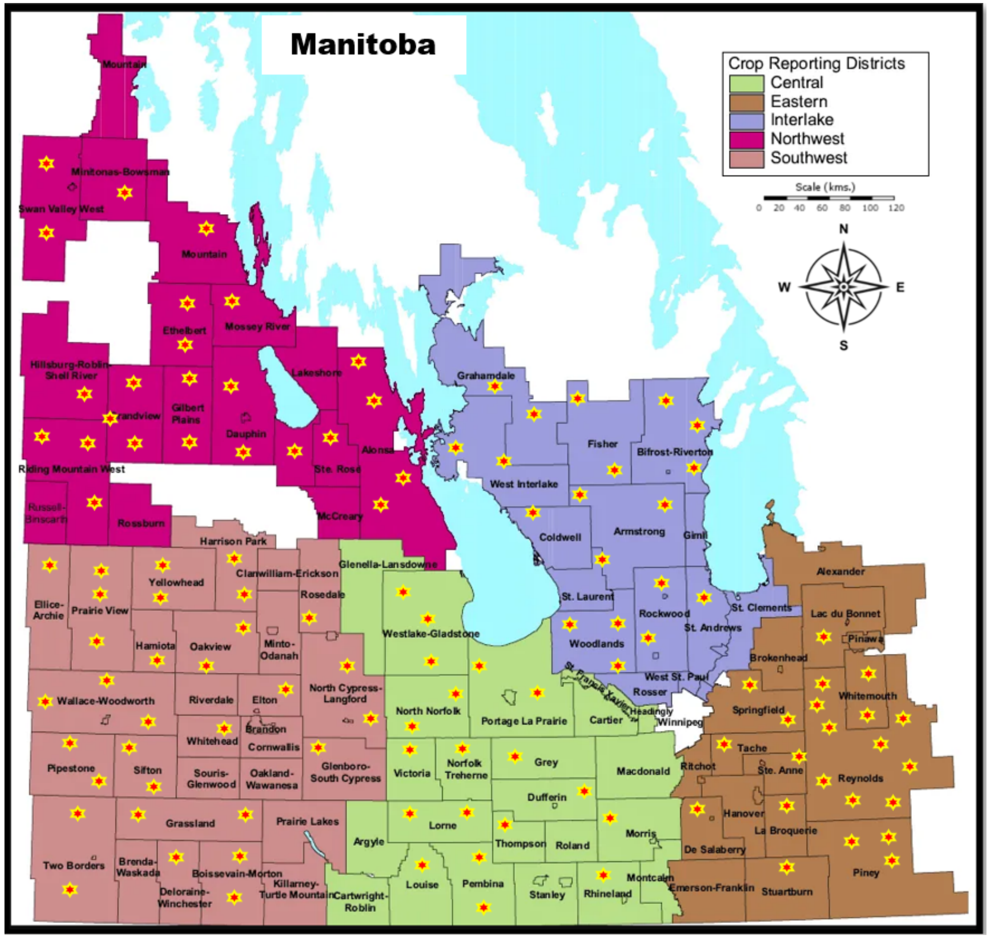

Figure S1. Geographical locations of surveyed oat fields in Manitoba from 2016 to 2018.
